# Preoperative embolization of highly vascular head and Neck Tumors and its impact on surgical outcome: A single-center experience

**DOI:** 10.12669/pjms.41.13(PINS-NNOS).13383

**Published:** 2025-12

**Authors:** Muhammad Naveed Majeed, Muhammad Fateen Rashed, Mohsin Zaheer, Asif Bashir, Qasim Bashir

**Affiliations:** 1 Dr. Muhammad Naveed Majeed, MBBS, MS Neurosurgery. Fellow Neuroendovascular Surgery, Department of Neuroendovascular Surgery, Punjab Institute of Neurosciences, Lahore, Punjab, Pakistan; 2 Dr. Muhammad Fateen Rashed, MBBS, FCPS Neurology. Fellow Neuroendovascular Surgery, Department of Neuroendovascular Surgery, Punjab Institute of Neurosciences, Lahore, Punjab, Pakistan; 3 Prof. Dr. Mohsin Zaheer, MBBS [PB]; FCPS Neurology. Department of Neurology, Punjab Institute of Neurosciences, Lahore, Punjab, Pakistan; 4 Prof. Dr. Asif Bashir, MBBS (PB); MD, FAANS, FACS (USA) Diplomate American Board of Neurological Surgery Reconstructive Spine Surgery, Department of Neurosurgery, Punjab Institute of Neurosciences, Lahore, Punjab, Pakistan; 5 Prof. Dr. Qasim Bashir, MBBS [PB]; MD [USA]; FSVIN [USA], Cast Certified Neuroendovascular Surgeon [USA]. Department of Neuroendovascular Surgery, Punjab Institute of Neurosciences, Lahore, Punjab, Pakistan

**Keywords:** Embolization, Therapeutic, Head and Neck Neoplasms, Preoperative Care, Treatment Outcome

## Abstract

**Objective:**

To evaluate how pre-operative trans-arterial embolization (TAE) affects the surgical outcomes of patient undergoing excision of vascular head and neck tumors.

**Methodology:**

This retrospective observational study was conducted at Department of Neuroendovascular Surgery, Punjab Institute of Neurosciences, Lahore, Pakistan from February 2024 to January 2025. It is a non-probability based consecutive case series, including 16 patients in accordance with the inclusion criteria.

**Results:**

Out of the 16 Patients enrolled in the study, 14 were male and 2 females with a mean age of 19.7±7.71 Years (Range; 14-37 Years). Except two patients with meningioma, all of the patients had JNA. The mean time between surgical excision and embolization was 38.6±18.9 hours, with most operations performed within 24–48 hours. Devascularization was graded as good in 12 patients (75%), fair in 2 (12.5%), and poor in 2 (12.5%). Mean operative times were 4.3 hours for good, 5.0 hours for fair, and 6.0 hours for poor devascularization. Corresponding ICU stays were 30, 48, and 72 hours, while ward stays averaged 2.2, 2.5, and 3.0 days, respectively. Better devascularization correlated with shorter surgery duration, reduced ICU time, and earlier discharge.

**Conclusion:**

Preoperative TAE is effective for highly vascular head and neck tumors, enabling shorter surgeries, easier debulking, and reduced blood loss. Polyvinyl alcohol (PVA) particles provide clear tumor margins and satisfactory devascularization.

## INTRODUCTION

Reduced tumor vascularity and intraoperative blood loss may result from preoperative embolizing of highly vascular tumors, ultimately facilitating surgical resection.^1^-^3^ Juvenile Nasopharyngeal Angiofibroma (JNA) and Meningiomas are one of the Head and Neck tumors which presents a clinical challenge being highly vascular and their proximity to basic neurologic structures.^4^ Meningiomas with histological confirmation make for 39% of all primary brain tumors and 54.5% of all non-malignant brain tumors, with an incidence of 9.12/100,000 people.^5^

The idea that preoperative embolization of meningiomas is a beneficial adjuvant in meningioma treatment is supported by the meta-analysis. This technique cuts lowers surgical time and blood loss during meningioma resection.^6^ JNAs are unencapsulated, well circumscribed polypoid masses that occurs almost exclusively in adolescent males, usually situated within Nasal Cavity, Sphenopalatine foramen and Nasopharynx. A multimodality approach that includes radiographic imaging to identify the feeder vessels of these highly vascular tumors and delineate this blood supply prior to surgical interference is essential to prevent any potentially dangerous situations and to optimize maximal safe resection, especially considering the significant blood loss that occurs during these procedures.^7^

Embolization can be trans-tumoral or trans-arterial. The trans-tumoral approach is often preferred for accessible tumors like JNA and hemangiomas due to its minimally invasive nature, precise targeting, and ability to induce tumor necrosis, leading to size reduction and improved outcomes.^8^ However, the trans-arterial route is suitable for nearly all lesions, including meningiomas, offering greater safety and precision while minimizing the risk of non-target embolization often associated with trans-tumoral injections.^9^ Preoperative embolization is underreported in public sector settings, especially in low-resource environments. Most data come from high-income centers, leaving a gap in the literature. This study shares our single-center experience with trans-arterial embolization (TAE) in highly vascular head and neck tumors and evaluates its effect on operative time, blood transfusion needs, and hospital stay.

Our study also aimed to highlight key technical aspects that determine the extent of devascularization and to explore ways to optimize these outcomes. Understanding the angiographic, anatomical, and procedural factors influencing the degree of devascularization can help refine embolization strategies, improve surgical conditions, and further reduce perioperative morbidity.

## METHODOLOGY

A retrospective observational study was conducted at the Department of Neuroendovascular Surgery, Punjab Institute of Neurosciences, Lahore, Pakistan from February 2024 to January 2025. The sampling technique used was non-probability consecutive sampling. A total of 16 patients who met the eligibility criteria were included.

### Ethical Approval:

The study was approved by the Ethical Review Board of Punjab Institute of Neurosciences under the reference no. 1900/IRB/PINS/Approval/2024, dated 10-09-2024.

### Inclusion Criteria:

Participants were eligible if they met the following criteria:

- Patients diagnosed with a highly vascular space-occupying lesion (SOL) based on clinical and radiological findings.
- Patients that could safely undergo preoperative embolization (free of the exclusion criteria).
- Tumors deemed suitable for surgical resection following preoperative embolization.

### Exclusion Criteria:

Participants were excluded if they met any of the following:

- Contraindications to embolization such as prior ischemic stroke, significant coagulopathy, or severe vasculitis.
- Known allergy to contrast agents.
- Current use of fibrinolytic or anticoagulant drugs at the time of embolization.
- Receipt of preoperative adjuvant therapy for the index tumor.

The embolization procedure was performed by an experienced interventional neurologist using high-resolution biplane DSA. Steroid coverage was given when indicated. A 5F micropuncture sheath set was used to access the right common femoral artery, followed by selective catheterization of intracranial vessels. For JNA cases, embolization was performed through extracranial vessels, typically branches of the external carotid artery, using a guide catheter (Hink 4fr) or microcatheter (Echelon 10; Covidien) and polyvinyl alcohol particles (150–350 µm). Catheters were inserted carefully to reduce lesion blood supply, and post-procedure hemostasis was achieved by manual compression. Following embolization, patients underwent ENT/neurosurgical procedures based on lesion location, vascularity, and embolization effect, with the goal of maximal safe lesion resection and complication prevention. In cases where ICA supply was embolized, the feeder arose from the meningohypophyseal trunk, and embolization was performed with extreme caution to avoid non-target cerebral ischemia. All surgical resections following embolization were performed by the same senior neurosurgeon/ENT surgeon team to maintain consistency in operative technique and assessment.

### Data analysis technique:

Data were collected from hospital records. Preoperative data included demographics, tumor type, vascular supply, arterial feeders, radiologic and clinical presentation, and embolic agents used. Operative data included time between embolization and surgery, duration of surgery, ease of lesion dissection, assessed intraoperatively by the operating surgeon and classified as easy, fair, or difficult. This was a subjective assessment based on tumor consistency and necrosis: “Easy” – predominantly necrotic and friable, easily suction able; “Fair” – mixed soft and firm components requiring both suction and sharp dissection; “Difficult” – predominantly firm, requiring sharp dissection or ultrasonic aspiration. Postoperative data included ICU stay, ward stay, pathological diagnosis, and tumor consistency. The extent of devascularization was assessed on post-embolization digital subtraction angiography (DSA) runs and categorized as follows: Good – ≥70% reduction in tumor blush; Fair – 30–69% reduction; Poor – <30% reduction. Data were analyzed using Statistical Package for Social Sciences (SPSS) version 27. P-values were calculated with < 0.05 considered significant using Chi-square and independent sample tests.

## RESULTS

Of the 16 patients enrolled in the study, 14 were male and two were female with a mean age of 19.7±7.71 years (range: 14–37 years) (Table-I). Except for two patients with meningioma, all others were diagnosed with juvenile nasopharyngeal angiofibroma. All nasopharyngeal angiofibroma patients presented with epistaxis, while the meningioma patients presented with bilateral vision deterioration (Table-I).

**Table-I:** Gender, Tumor Type, and Vascular Supply of Patients

Parameter	Category	Number	Percentage (%)
Gender	Male	14	87.5
	Female	2	12.5
Tumor Type	Juvenile Nasopharyngeal Angiofibroma	14	87.5
	Meningioma	2	12.5
Vascular Supply	Right ECA (IMAX branches)	6	37.5
	Left ECA (IMAX branches)	7	43.75
	Bilateral ECA (IMAX branches)	1	6.25
	Right ICA + Right ECA (IMAX branches)	2	12.5

Legend: IMAX – Internal Maxillary Artery; ECA – External Carotid Artery; ICA – Internal Carotid Artery.

All embolization procedures were performed transarterially and completed uneventfully, using polyvinyl alcohol particles as the embolic agent. Post-embolization runs consistently demonstrated significant reduction in tumor enhancement, although the degree of devascularization varied (Table-II).

**Table-II:** Intraoperative Parameters and Statistical Analysis

Parameter	Category	Number (%)	p-value (Fisher’s Exact Test)
Extent of Devascularization	Good	12 (75%)	1.000
	Fair	2 (12.5%)	
	Poor	2 (12.5%)	
Lesion Dissection & Debulking	Easy	12 (75%)	1.000
	Fair	2 (12.5%)	
	Difficult	2 (12.5%)	
Blood Transfused	1 Unit	12 (75%)	0.011
	2 Unit	2 (12.5%)	
	3 Unit	2 (12.5%)	

The mean time between embolization and surgical excision was 38.6±18.9 hours, and all patients underwent surgery within 24–48 hours. A small number of cases (2) exceeded 48 hours due to logistical or clinical considerations. The mean operative time was 4.6±1.1 hours, with only two surgeries exceeding 5 hours. Twelve patients (75%) had good devascularization, while two each had fair and poor outcomes. Lesion dissection was reported to be easy in 75%, fair in 12.5%, and difficult in 12.5% of cases. Blood transfusion requirement was 1 unit in 75%, 2 unit in 12.5%, and three unit in the remaining 12.5% (Table-II).

In the two cases rated as “Poor” devascularization, DSA revealed multiple arterial feeders, including contributions from the contralateral external carotid artery and small pial branches in addition to the ipsilateral internal maxillary artery (IMAX). These additional feeders could not be embolized due to technical limitations such as vessel tortuosity and the risk of non-target embolization to eloquent brain territories. In one case, microcatheter navigation into the pial feeders was not feasible without risking vessel injury. The other case had dual supply from the IMAX and ophthalmic artery branches, where embolization risked compromising ocular perfusion.

Following surgery, all patients were transferred to the surgical ICU with a mean ICU stay of 30±6 hours; 12 patients stayed 30 hours, two stayed 48 hours, and two stayed 72 hours. Patients were then shifted to the neurosurgical/ENT ward, where the mean ward stay was 2.4±0.6 days (Table-III). The mean time between embolization and surgical excision was 38.6 ± 18.9 hours, with most patients undergoing surgery within 24–48 hours. When stratified by devascularization category (Table-III), the mean operative time was 4.3 ± 0.9 hours for the Good group, 5.0 ± 0.7 hours for Fair, and 6.0 ± 0.0 hours for Poor devascularization. The corresponding mean ICU stays were 30 ± 0 hours, 48 ± 0 hours, and 72 ± 0 hours, respectively. Mean ward stays were 2.2 ± 0.4 days, 2.5 ± 0.0 days, and 3.0 ± 0.0 days, respectively. Kruskal–Wallis testing showed statistically significant differences between groups for all parameters (p < 0.05).

**Table-III:** Mean Times and Correlation with Devascularization

Parameter	Good (n=12) Mean ± SD	Fair (n=2) Mean ± SD	Poor (n=2) Mean ± SD	p-value (Kruskal–Wallis)	Spearman p	Spearman p
Operative Time (hrs)	4.3 ± 0.9	5.0 ± 0.7	6.0 ± 0.0	0.013	-0.73	0.0013
ICU Stay (hrs)	28 ± 3	48 ± 0	72 ± 0	<0.001	-1.00	<0.001
Ward Stay (days)	2.2 ± 0.4	2.5 ± 0.0	3.0 ± 0.0	<0.001	-1.00	<0.001

No statistical test was applied to “Time between Embolization and Surgery” as it followed a standardized protocol with minimal variation across patients. However, there was a strong negative correlation between devascularization quality and operative time (ρ = -0.73, *p* = 0.0013), indicating shorter surgeries with better devascularization. ICU and ward stay also showed perfect inverse correlation with devascularization grade (*p* < 0.001), further affirming the efficacy of pre-operative embolization in improving surgical outcomes.

## DISCUSSION

This focused experience evaluated the safety and efficacy of preoperative embolization in surgical resection of Highly Vascular Intracranial tumours and JNA in South Asian population. A total of 16 tumours, predominantly male with a mean age of 19.2 underwent embolization followed by surgical resection. Our findings demonstrated a high rate of good tumor devascularization with a well-defined line of cleavage. This facilitated easier dissection and maximum debulking, reduced blood loss, and shortened operative time. It also contributed to fewer post-procedural complications and a favorable postoperative recovery. Our results are in line with local Pakistani studies, which also report high technical success of TAE in JNA and meningiomas, with reduced intraoperative blood loss and favorable surgical outcomes.^10^

In our setting, the surgical candidacy post-TAE was determined by the degree of devascularization achieved, tumor accessibility, and the patient’s overall clinical stability. All patients in this series proceeded to surgery as planned following TAE, which reflects our protocol of performing surgery within 24–48 hours, provided there were no new neurological deficits or significant post-procedural complications. This high conversion rate to surgery contrasts with some reports from similar low-resource settings, where limited access to hybrid operating suites or neurointerventional teams resulted in 10–20% of patients experiencing delayed or canceled resections due to incomplete embolization or peri-procedural complications.^11^

**Fig.1:**
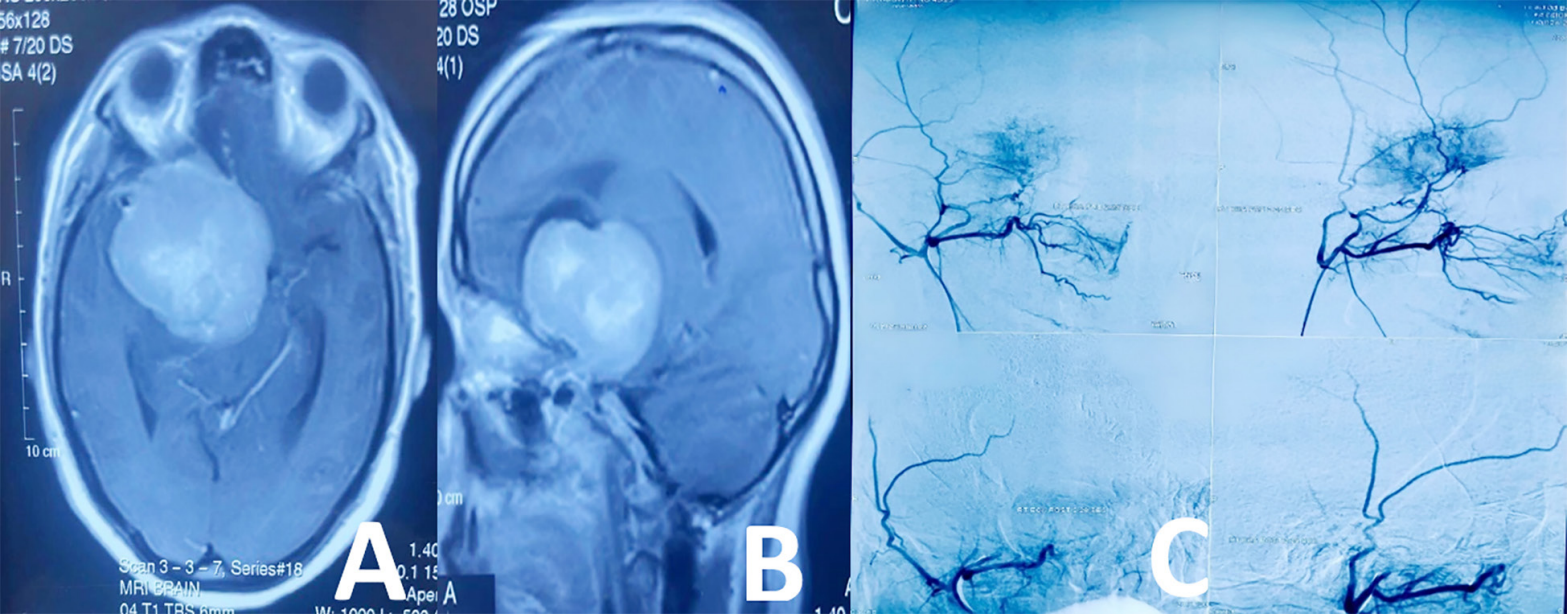
Case 1.: Skull Base Meningioma, A: Axial T1 with Contrast, B: Sagittal T1 with Contrast, C: Pre & Post PVA embolization DSA

**Fig.2:**
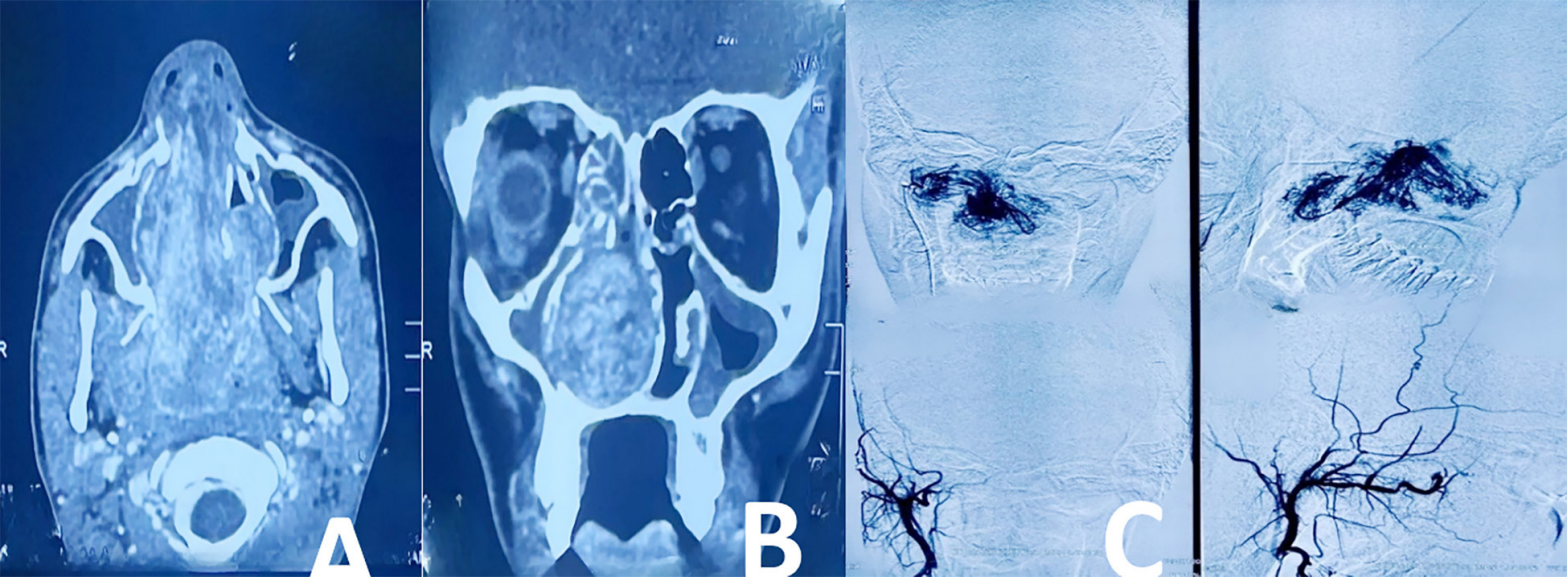
Case 2.: Juvenile Nasopharyngeal Angiofibroma, A: Axial T1 with Contrast, B: Coronal T1 with Contrast, C: Pre & Post PVA embolization DSA.

Our findings are within the broader literature; many comparative studies reinforced the superiority of embolization in surgical procedures involving Hyper vascular tumours. It is no secret that close proximity of these tumours makes their excision very difficult even for the most experienced of surgeons. Creating tumor devascularization and increasing tumor necrosis at the precapillary level to minimize intraoperative bleeding, enabling a more radical and practical excision, and lowering the likelihood of recurrence are the primary goals of preoperative embolization of tumors. Since the effect of embolization eventually fades due to the re-revascularization phenomena of skull-base tumors, surgery should only be performed within seven days of embolization and only after 24 hours at the latest. Because the external carotid artery typically provides the vascular supply for meningiomas, embolization can be regarded as a low-risk operation.^12^ Not only for Nasopharyngeal Angiofibroma and Meningiomas which were studied in this cohort Fig.1 and Fig.2, Embolization has been evaluated and proven to enhance surgical precision, reduce morbidity and improve prognosis for various types of Hypervascular Lesions including Hemangiopericytoma, Hemangioblastoma (HGBs), Glomus Jugulare and other paragangliomas. Carotid Body Tumors in particular have been analysed in many studies, including but not limited to single case reports that often-involved different approaches to the procedure in question. Catapano et al. noted the excellent devascularization of skull base paragangliomas and facilitates resection in about 80% of the patients post TAE.^13^ Similarly, success of TAE for 17 HGBs manifested as gross total resection in 91% and improved, stable functional status in 75% of patients was observed by Moscovivi et al.^14^

Schartz et al. conducted a systematic review and meta-analysis to evaluate the variations in the results of surgically removing a meningioma in a group of two patients, one with and one without TAE. It was discovered that during follow-ups, the TAE group had a considerably lower chances ratio of severe surgery-related problems and a greater odds ratio of post-operative functional independence.^15^

Our findings align with regional reports from South Asia, where TAE has demonstrated high technical success but variable impact on operative parameters depending on feeder anatomy and embolic material availability.^16^ For example, a tertiary center study from India reported good (>70%) devascularization in 72% of juvenile nasopharyngeal angiofibroma, comparable to our 75%, though they noted higher rates of incomplete embolization in meningiomas supplied by multiple feeders, similar to our two “Poor” cases. Internationally, centers in high-resource settings report good devascularization rates exceeding 85–90%, often attributed to wider availability of liquid embolics like Onyx and advanced microcatheters. This highlights the gap in procedural tools between resource-limited and advanced centers, which can directly influence surgical ease and blood loss reduction.

Commonly used materials for TAE include Onyx, PVA, and Gel Foam. Onyx and PVA provide good devascularization with clear margins, yielding superior resection outcomes in about 80% of cases. Microfibrillar collagen is another effective option, capable of penetrating small end arteries and shown to have long-term benefits in previous studies.^17^ With that said, embolization does come with some complications including cranial nerve palsies, damage to healthy tissue following nonselective targeting, neurologic deficits from the migration of the embolic agents as well catheter related trauma and puncture site infections. In one study, three patients experienced mild catheter-related complications, and one patient experienced temporary hemiparesis, for a 13.4% overall procedural complication rate. One of the seven patients who had embolization for skull base paragangliomas had facial palsy, according to Catapano et al.^13^ For nasopharyngeal angiofibroma, Kothari et al. found a TAE-related complication rate of 3.16%. These complications although rare, should be considered when taking informed consent from the patient. Also note that the time frame of 24-48 hours between embolization and surgical resection, as practiced in this study is optimal for equilibrating the benefits of reduced bleeding with the risks of embolization related complication.^7^

Postoperative complications in our cohort were minimal. This is consistent with other local series, which also report low morbidity when embolization is performed selectively and surgery is undertaken promptly. In contrast, some international series report higher complication rates, partly due to inclusion of more complex skull base lesions and use of aggressive embolization techniques that may increase non-target ischemia risk. The relatively favorable outcomes in our study may therefore reflect both careful patient selection and the avoidance of high-risk feeders in complex anatomy.^18^

Even though two cases of meningioma have been treated in this study, many other cohorts have centered around them to evaluate the surgical outcomes as long as one year, reporting improved complains in about 70% of patients and effectively reducing the perioperative blood loss, as judged by Al-Mufti et al. by the change in pre- and post-procedural hemoglobin and hematocrit levels.^19^

### Limitations of the study:

The study has several limitations inherent to its single-center, retrospective study design, small sample size and lack of heterogeneity amongst the tumour types limits the generalizability of our results. Additionally, it’s a single arm study with no control group of patients that did not undergo embolization, hence preventing us from making a fair comparison and to be able to definitively attribute improvements in surgical outcomes wholly to the embolization procedure. Due to the lack of long-term follow-ups’, long term outcomes such as recurrences or postoperative complications were not assessed. Moreover, the study design was observational which are lower in hierarchy as compared to randomized controlled trials which are more accurate.

### Clinical recommendations:

Future studies with larger sample sizes, longer follow-up periods, and multi-center collaboration are needed to validate these findings and explore the long-term outcomes of postoperative embolization in managing highly vascular head and neck tumors.

## CONCLUSION

Transarterial embolization of highly vascular tumour is an effective preprocedural technique to facilitate maximal surgical excision with minimal hemorrhage and the need for blood transfusions. Although complications occur, Transarterial PVA particles embolization provides satisfactory devascularization and well defines the line of demarcation.

### Authors Contribution:

**MNM, MF:** Data acquisition, analysis and interpretation and drafted the manuscript.

**QB, MZ, AB:** Concept and design of the study, critically reviewed and supervised the study.

All authors have approved the final version to be published and agreement to be accountable for all aspects of the work in ensuring that questions related to the accuracy or integrity of any part of the work are appropriately investigated and resolved.

## References

[1] Ampie L, Choy W, Lamano JB, Kesavabhotla K, Kaur R, Parsa AT (2016). Safety and outcomes of preoperative embolization of intracranial hemangioblastomas:a systematic review. Clin Neurol Neurosurg.

[2] Alberione F, Iturrieta P, Schulz J, Masenga G, del Giudice G, Ripoli M (2010). Preoperative embolisation with absorbable gelatine sponge in intracranial meningiomas. Reply. Revista de Neurología.

[3] Satyarthee GD, Kumar S (2018). Concern and usefulness of intratumoral injection of ethyl alcohol for devascularization of intracranial tumors. Turk Neurosurg.

[4] Duffis EJ, Gandhi CD, Prestigiacomo CJ, Abruzzo T, Albuquerque F, Bulsara KR (2012). Head, neck, and brain tumor embolization guidelines. J Neurointerventional Surg.

[5] Pikis S, Mantziaris G, Dumot C, Xu Z, Sheehan J (2023). Stereotactic radiosurgery for intracranial meningiomas. Neurosurg Clin.

[6] Chen L, Li D-h, Lu Y-h, Hao B, Cao Y-q (2019). Preoperative embolization versus direct surgery of meningiomas:a meta-analysis. World Neurosurgery.

[7] Kothari DS, Linker LA, Tham T, Maroda AJ, McElfresh JM, Fastenberg JH (2023). Preoperative embolization techniques in the treatment of juvenile nasopharyngeal angiofibroma:a systematic review. Otolaryngology–Head and Neck Surg.

[8] Hong CS, Marianayagam NJ, Morales-Valero SF, Barak T, Tabor JK, O'Brien J (2023). Vascular steal and associated intratumoral aneurysms in highly vascular brain tumors:illustrative case. J Neurosurg:Case Lessons.

[9] Hohenstatt S, Angileri SA, Granata G, Paolucci A, Ierardi AM, Carrafiello G (2021). Resorbable purified porcine skin gelatin cross-linked with glutaraldehyde spheres for preoperative embolization of carotid body paraganglioma. Acta Bio Medica:Atenei Parmensis.

[10] Shamim AA, Ghias K, Khan MJ (2013). Juvenile nasopharyngeal angiofibroma:experience at a tertiary care centre in Pakistan. J Pak Med Assoc.

[11] Morgan R, Haslam P, McCafferty I, Bryant T, Clarke C, McPherson S (2024). Provision of interventional radiology services 2023. Cardiovasc Intervent Radiol.

[12] Moscote-Salazar LR, Dolachee AA, Narvaez–Rojas A, Al–Saadi HA, Najim AA, Abdulreda AK (2019). Preoperative embolization of skull–base tumors:Indications, utility, and concerns. J Acute Dis.

[13] Catapano JS, Almefty RO, Ding D, Whiting AC, Pines AR, Richter KR (2020). Onyx embolization of skull base paragangliomas:a single-center experience. Acta Neurochirurgica.

[14] Moscovici S, Candanedo C, Spektor S, Cohen JE, Kaye AH (2022). Solid vs. cystic predominance in posterior fossa hemangioblastomas:implications for cerebrovascular risks and patient outcome. Acta Neurochirurgica.

[15] Schartz D, Furst T, Ellens N, Kohli GS, Rahmani R, Akkipeddi SMK (2023). Preoperative embolization of meningiomas facilitates reduced surgical complications and improved clinical outcomes:a meta-analysis of matched cohort studies. Clin Neuroradiol.

[16] Butt AS, Hamid S, Butt N, Sharif F, Haq TU, Jafri W (2016). Is transarterial embolization a valuable treatment option for spontaneous rupture of hepatocellular carcinoma:experience from a tertiary care hospital of South-Asia. Hepatoma Res.

[17] Kaufman S, Kumar A, Roland J, Harrington D, Barth K, Haller Jr J (1980). Transcatheter embolization in the management of congenital arteriovenous malformations. Radiology.

[18] Ylimartimo AT, Nurkkala J, Koskela M, Lahtinen S, Kaakinen T, Vakkala M (2023). Postoperative Complications and Outcome After Emergency Laparotomy:A Retrospective Study. World J Surg.

[19] Al-Mufti F, Gandhi CD, Couldwell WT, Rybkin I, Abou-Al-Shaar H, Dodson V (2023). Preoperative meningioma embolization reduces perioperative blood loss:a multi-center retrospective matched case-control study. Br J Neurosurg.

